# Midterm Results of Cementless Total Knee Arthroplasty: A Retrospective Case Series

**DOI:** 10.2174/1874325001812010196

**Published:** 2018-06-14

**Authors:** Radosław Stempin, Kacper Stempin, Wiesław Kaczmarek, Julian Dutka

**Affiliations:** 1 Department of Orthopedic & Trauma Surgery, J. Strus Memorial (Multispecialistic City) Hospital, Poznan, Poland; 2 Department of Orthopedic & Trauma Surgery, Westallgäu Clinic, Wangen, Germany; 3 Department of Orthopedic Surgery, Promienista Clinic, Poznan, Poland; 4 Department of Orthopedic & Trauma Surgery, S. Zeromski Memorial Hospital Cracow, Poland

**Keywords:** Osteoarthritis knee, Cementless total knee arthroplasty, Fixation, Bone matrix quality, Bone hardness test, Biocompatible coated materials

## Abstract

**Background:**

There is an ongoing debate about whether to use cementless or cemented fixation for Total Knee Arthroplasty (TKA).

**Objective:**

The study aimed to assess midterm survivorship of the Vanguard cementless system, and to demonstrate the utility of the Bone Hardness Test (BHT) for the selection of cementless fixation TKA.

**Methods:**

From September 2009 through November 2014, 123 total knee arthroplasties were completed, with cementless Vanguard Cruciate Retaining TKA in 110 knees (102 patients) and cemented Vanguard in 13 cases (12 patients). Implant fixation was based on intraoperative assessment of posterior cruciate ligament stability, bone quality, and BHT. All patients with a cementless Vanguard implant were eligible for this retrospective study. Preoperative and postoperative Knee Society Score and Western Ontario and McMaster Universities Osteoarthritis Index were obtained. Standardized standing anteroposterior and lateral radiographs were taken.

**Results:**

Three patients (4 TKAs) were lost to follow-up. The mean follow-up time was 5.5 ± 1.4 years. All scores significantly improved postoperatively. No radiographic failures were observed. Five-year implant survival, with revision of any component for any reason as an endpoint, was 97.2% (95% confidence interval, 91.7 - 99.1%). Five-year survival with revision for aseptic loosening was 100%. Only one knee required revision due to an isolated unrelated bearing exchange, and two additional knees required secondary resurfacing of the patella for retropatellar pain.

**Conclusion:**

Good midterm results were obtained with the cementless Vanguard Cruciate Retaining TKA for the treatment of osteoarthritis. The Bone Hardness Test appears to be an effective way to determine the selection of cementless TKA.

## 1 INTRODUCTION

Ever since the advent of cementless systems for Total Knee Arthroplasty (TKA), there has been discussion over when to choose this newer modality over conventional cemented fixation for TKA [1-4]. Cemented systems have created issues with cement-bone interface degradation and implant loosening, so the cementless TKA was designed to promote osseointegration and offer long-term fixation – especially in young and active patients [1, 5].

However, osteoconductive coatings reduce micromotions and improve osseointegration, both in young patients and in patients over the age of 75, without any significant differences between these age groups. The results of cementless TKA in elderly patients are clinically comparable with those in younger patients, with good outcome reported at five years in both age groups [6].

Other reported advantages of cementless TKA are that it is potentially time-saving during surgical implantation; it reduces ischemia time; and allows an easier, more bone-preserving, revision in the event of failure [7]. It has been argued that cementless knee implants are more expensive than cemented, but their shorter operative time allows for reduced costs associated with surgery [8].

There is a growing number of patients younger than 65 undergoing TKA, and there is concern that primary implants will not last for a lifetime, especially in these younger and more active patients [9].

The choice between cemented and cementless TKA is not straightforward, and the selection criteria is widely debated in the literature [10-12]. Good bone matrix quality seems to be critical to minimize the risk of migration and aseptic loosening of the tibial component. In unicompartmental arthroplasty (UKA), the Bone Hardness Test (BHT) has been proposed as a simple and useful way to assess the eligibility of patients for cementless unicompartmental replacement [13]. The first author of this paper applied the same principles for the present study, which was designed to assess midterm outcome with the cementless Vanguard TKA, applying the BHT as a selection criterion for patient eligibility.

## 2 MATERIALS AND METHODS

At our clinic, 123 total knee arthroplasties were completed, implanting the cementless Vanguard Cruciate Retaining TKA (Zimmer Biomet, Inc, Warsaw, IN, USA) in 110 knees (102 patients) and the cemented version of the Vanguard in 13 knees (12 patients), between September 2009 and November 2014. The choice of cementless implant was based on intraoperative assessment of posterior cruciate ligament stability, visual inspection of bone quality, and on the BHT, which was determined by exerting pressure with a thumb or index finger on the surface of the tibial bone following resection of the tibia. If the resected surface deflected when pressure was applied to the bone (*i.e.*, if the thumb delved into the bone tissue), the bone hardness was deemed insufficient for primary stability of the cementless implant, and a cemented TKA implant was used.

Both the cementless femoral and the cementless tibial components are constructed of a chromium-cobalt alloy covered with a porous, pure titanium plasma spray coating to promote osseointegration. The femoral component has an extended trochlear groove that allows the patella to maintain contact with the femoral component during flexion. No updates to the design have been made since the system was introduced to our clinic.

We conducted a retrospective study of the cementless Vanguard system in a consecutive series of patients. Osteoarthritis was the primary indication for TKA in all patients, comprising 82 (80.4%) females and 20 (19.6%) males with an average age of 72.7 ±7.1 (range, 59 - 81) years.

For the surgery, all patients were placed in a supine position, and most patients (n = 97) received a spinal anesthesia, with only 5 patients undergoing general anesthesia. A tourniquet and an anteromedial parapatellar approach was used in all cases by a single, experienced surgeon (R.S.). The bone resections were performed accurately in order to avoid any gaps between the bone substrate and the prosthetic components. The sclerotic surfaces of the bones were removed completely. Minimal tibia bone was resected, since the strength of cancellous bone decreases with increasing depth of resection [14, 15].

Patients used perioperative prophylactic antibiotic therapy for three days. All patients received thrombosis prophylaxis with fraxiparine for 2 to 4 weeks, depending on the comorbidity status of the patient. On the first postoperative day, patients ambulated with full weight bearing with the assistance of a walker. Postoperative pain management consisted of morphine, tramadol, paracetamol, diclofenac sodium, pyralginum, and ketoprofen (IM). In the first few days after surgery, physical therapy was prescribed. On average, patients were discharged from the hospital on the fourth postoperative day.

Clinical and radiographic evaluation was completed preoperatively and at final follow-up. Functional assessment was determined by the Knee Society Score (KSS) [16] and with the Western Ontario and McMaster Universities Osteoarthritis Index (WOMAC) score [17]. Standardized standing anteroposterior and lateral radiographs were taken and analyzed for periprosthetic Radiolucent Lines (RLLs), evidence of component subsidence, focal osteolysis, and polyethylene wear. RLL measuring greater than 1mm in all zones or a change in implant location constituted a loss of biological fixation [1]. Definitive biological fixation was noted upon radiological evidence of the absence of RLLs between the implant and the bone at all radiographic zones of both prosthetic components [18].

In accordance with Polish law, ethics committee approval was not obtained, since the study was observational and did not involve changes to standard clinical practices.

### 2.1 Statistical Analysis

For all measured outcomes reported in the study results, all values were calculated as mean ± SD. Kaplan Meier analysis, with calculation of 95% Confidence Intervals (CI) was employed for survival analysis. Endpoints of interest included revision of any component for any reason and revision of any component for aseptic loosening.

## 3 RESULTS

Two patients (2 TKAs) died from unrelated causes during the course of the study and one patient (2 TKAs) was lost to follow-up. Hence, 106 knees (99 patients), were available for clinical follow-up assessment. The mean follow-up time was 5.5 ± 1.4 (range, 3.2 – 8.0) years.

Overall, one knee (0.9%) required revision for an isolated bearing exchange. A secondary resurfacing of the patella was performed in two knees (1.9%) for retropatellar pain. An additional patient experienced a traumatic tibial fracture, but was not operated on due to a poor general health condition. His pre-accident scores were comparable to the overall population mean.

**Fig. (1):**
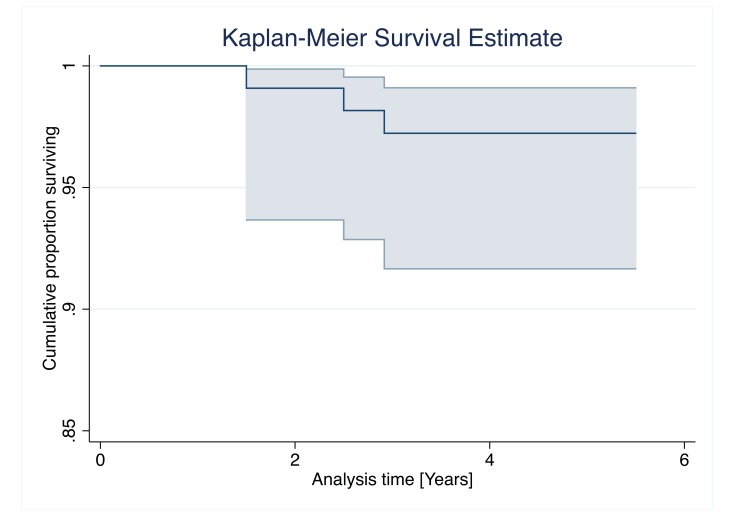
Implant survival with revision for any cause as endpoint of interest.

Five-year implant survival, with revision of any component for any reason as the endpoint of interest, was 97.2% (95% CI, 91.7 - 99.1%) (Fig. **1**). Five-year survival with revision for aseptic loosening as endpoint of interest was 100%.

The mean KSS clinical score increased from 36 ± 12 preoperatively to 88 ± 10 at follow-up evaluation, and the function score improved from 35 ± 9 to 74 ± 13. The mean WOMAC score increased from 38 ± 2 preoperatively to 78 ± 6 at follow-up assessment. With the exception of the previously mentioned patients who required secondary patella resurfacing, no patients with anterior knee pain in the non-resurfaced patella were observed. There were no patients with subluxation or dislocation of the patella.

**Fig. (2):**
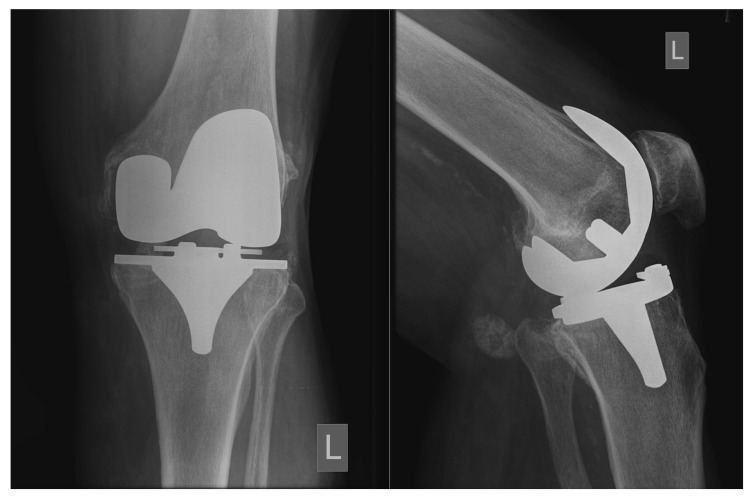
Anteroposterior (left) and lateral (right) radiographs showing the cementless Vanguard in a 78-year-old female with favourable clinical outcome 5 years postoperatively. No radiolucent lines are visible.

Radiographic assessment could be performed in 97 knees. We did not observe any radiographic failures on the tibia or femur at follow-up. In 9 out of 97 knees, RLLs were observed. RLLs were small (< 1 mm), incomplete, and non-progressive in all cases (Fig. **2**).

Additionally, no signs of polyethylene wear, subsidence of the tibial component or osteolysis of either the tibia or femur were observed. Osseointegration of both components was observed radiologically in all evaluated knees.

## 4 DISCUSSION

The Vanguard primary cementless, posterior cruciate-retaining TKA system without patella resurfacing yielded excellent clinical results with high survival in this retrospective clinical study. We noted 100% implant survival with implant loosening as the endpoint of interest. Two cases needed secondary patellofemoral replacement, but a revision of the well-fixed femoral and tibial components was not required. Preoperative bone quality has an important impact on the cementless fixation. A significant association between preoperative level of Bone Mineral Density (BMD) measured in the proximal tibia and migration of the tibial component has been established [19]. However, the objective measurement of the average BMD in the medial and lateral condyles in order to objectively determine the quality of the bone matrix is expensive compared with the BHT. Our simple assessment of bone substrate has resulted in good primary and secondary stabilization of cementless implants, which is supported by the fact that in the present series no subsidence and lift-off was seen.

Our study results are comparable to other cementless TKA systems, which reported similar survival rates [1, 18, 20, 21]. Results are also in line with studies performed on the cemented Vanguard. Faris *et al* reported a 10-year survival rate of 98.4% for the cemented Vanguard with revision for any reason as endpoint of interest [22]. Similarly, Kievit *et al.* reported a 6-year survival of 96.5% with revision for any reason as endpoint of interest, with secondary resurfacing of the patella performed in 1.7% of the patients [23].

A review of revision surgeries revealed the indications for the secondary procedures. In a meta-analysis by Pilling *et al*, 48 (6%) of 792 knees in the non-resurfacing group required surgical re-intervention because of anterior knee pain [24]. In that report, the randomized controlled trials had short follow-up, with the latest follow-up often being five years after the primary procedure. As in our study, the percentage of revision surgeries was lower (2%), and it was assumed that these revisions were likely due to the natural progression of patellofemoral arthritis rather than a factor such as trochlear groove design.

While cemented fixation remains the gold standard for many, there is an increased interest in uncemented fixation in TKA [25]. Studies support the choice of both implants. While a meta-analysis published by Gandhi *et al* found significantly higher implant survival for cemented compared with cementless TKA (with follow-up time ranging from 2 to 11 years) [26], a review by Mont *et al* concluded that cementless and cemented TKA had similar implant survivorship (odds ratio, 1.1; 95% confidence interval [CI], 0.62-2.00). At 10 years follow-up, the mean survival rate for cementless TKA was 95.6%, compared with 95.3% for cemented TKA [4].

Advanced design developments have contributed to favorable results with cementless TKA [27]. Using our study system, the femoral and tibial components had excellent radiologic results, and none of the 120 originally implanted knees showed loosening after more than 5 years. The validity of our study results is strengthened by the low rate of attrition of 1.6%.

A limitation of the present study is uncontrolled design; a randomized clinical study comparing cemented and cemented Vanguard would have been informative. Another study limitation was that we have not been able to provide scientific evidence for the merit of the BHT. More studies are warranted before this test can be generally recommended for use in clinical practice.

Our study supports the success of cementless components and Bone Hardness Test for primary TKA by orthopedic surgeons treating patients with osteoarthritis. As components and techniques continue to advance, cementless fixation is progressing towards the goal of becoming the new gold standard for TKA.

## CONCLUSION

In conclusion, this study demonstrates favorable outcome for the cementless Vanguard Cruciate Retaining TKA in the treatment of osteoarthritis of the knee. Our study results show good midterm survival of the system which is consistent with the results for other cementless TKA systems. In addition, the Bone Hardness Test may be a simple and useful way of assessing eligibility for cementless TKA. Long-term studies with larger sample sizes are warranted.
